# Comparison of accumulation and distribution of PEGylated and CD-47-functionalized magnetic nanoporous silica nanoparticles in an ***in vivo*** mouse model of implant infection

**DOI:** 10.1371/journal.pone.0321888

**Published:** 2025-05-02

**Authors:** Heidi Harting, Timo Herrmann, Nina Ehlert, Jessica Meißner, Nina Angrisani, Janin Reifenrath

**Affiliations:** 1 Hannover Medical School, Department of Orthopaedic Surgery, DIAKOVERE Annastift, Hannover, Germany; 2 Lower Saxony Center for Biomedical Engineering, Implant Research and Development (NIFE), Hannover, Germany; 3 Institute for Inorganic Chemistry, Leibniz University Hannover, Hannover, Germany; 4 Department of Pharmacology, Toxicology and Pharmacy, University of Veterinary Medicine Hanover, Foundation, Hannover, Germany; Advanced Materials Technology Research Institute, National Research Centre, EGYPT

## Abstract

**Introduction:**

Drug targeting using nanoparticles is a much-researched topic. Rapid interactions of nanoparticles with the host’s immune system and clearance from the circulation is a major problem resulting in non-satisfying accumulation rates in the desired region. The aim of the presented study was to compare organ distribution and implant accumulation of magnetic nanoporous silica nanoparticles (MNPSNP) functionalized with either Polyethylenglycol (PEG) or CD-47 *in vivo* in a mouse model of implant infection.

**Methods:**

Synthesis and functionalization of the magnetic core-shell nanoparticles is described. In the *in vivo* study, 32 mice were included and received an in staphylococcus aureus solution preincubated magnetic implant subcutaneously on the left and a nonmagnetic implant on the right hind leg. MNPSNP accumulation in the inner organs as well as on and around the implants was analyzed in dependence on the functionalization.

**Results:**

MNPSNP were successfully functionalized with PEG or CD-47. *In vivo,* unexpectedly both nanoparticle variants accumulated mainly in liver and spleen. In the tissue, surrounding the implants higher nanoparticle accumulation was seen in areas with more severe signs of inflammation Nanoparticles were detectable on both implant materials, but accumulation rate was very low.

**Conclusion:**

Although various literature describes higher accumulation rates for nanoparticles functionalized with CD-47 in target areas and a reduced accumulation in liver and spleen, this could not be shown within this study. Possible instability or rapid agglomeration of the particles are conceivable reasons. Higher accumulation rates in areas with more severe signs of inflammation indicate that inflammatory cells might be essential for the delivery of nanoparticles into inflamed regions.

## Introduction

Considering the continuously aging society in high-income countries and the rising total number of arthroplasties [1,2], it is not surprising that the number of periprosthetic joint infections (PJI) is also on the rise [1,3,4]. Requiring long hospitalization periods, long-term antibiotics and often additional revision surgery, PJI is a high-cost public health problem [5]. In view of rising antibiotic resistance in bacteria as well as the resource intensive and expensive therapy, new strategies for the treatment of implant infections are urgently needed. Magnetic nanoparticles have a number of useful properties that make them ideal candidates for delivering antibiotics directly to an implant colonized with a bacterial biofilm.

Using nanoparticles (NPs) as a delivery vehicle for drugs and other cargo is an approach that has aroused enormous interest over the past decades. Polymeric, lipid or metal-based nanoparticles are only some examples of nanoformulations already described in literature [6–8]. The characteristics of magnetic and porous nanoparticles (MNPs) including small size, high surface-to-volume ratio, chemical stability, electromagnetic properties, biocompatibility and biodegradability [9–11] make them ideal candidates for a variety of applications. Targeted drug and gene delivery [12,13], magnetic hyperthermia [14], magnetic resonance imaging (MRI) [15] as well as biosensing [16], and bioseparation [17] are some of the main areas of application.

Drug targeting in general is the ability of a drug to selectively and quantitatively accumulate in a target organ or tissue [18]. Advantages are a simplification of drug administration protocols, a reduction of the overall amount of an administered drug, and an increase of the drug concentration at a specific target site without negative side effects [18]. Although drug delivery systems using nanoparticles are a highly researched field, recognition of the particles by cells of the mononuclear phagocytose system (MPS) and rapid clearance from the circulation is still one of the major problems [19]. To obtain longer circulation times various strategies were tested. Different approaches are 1) temporary exhaustion of the phagocytic capacity of Kupffer cells 2) induction of a transient depletion or elimination of macrophages or 3) functionalization of NPs to protect them from rapid recognition by the MPS [20]. Different studies also investigating magnetic nanoparticles [21,22] used macrophage depletion as an approach to prolong the circulation time of the particles. Injection of clodronate-loaded liposomes resulted in apoptotic cell death of monocytes, liver, and spleen macrophages. Although significant effects were described, the treatment had possible massive side effects [21,22].

Physicochemical characteristics like size, shape, surface properties, and material’s deformability/rigidity have an impact on the particle pharmacokinetics in the blood, as extensive research revealed [23,24]. Because of that and the disadvantages of the first two strategies, modification and functionalization are the most researched approach to extend the circulation time of nanoparticles. Some examples of molecules used to modify the surface of nanoparticles are small proteins, peptides, antibodies, aptamers, and oligosaccharides [25]. Human albumin for example can be used on NP surfaces to reduce toxicity and achieve active targeting at the same time [26].

A very common method to enhance biocompatibility and generate so-called stealth nanoparticles is the functionalization with polymers like polyethyleneglycol (PEG). A study showed that the biocompatibility of Fe_3_O_4_ and SiO_2_ NPs was increased when PEG was added to the surface up to high concentrations of 200 µg/ml with neural stem cells. In contrast, the bare particles caused a viability reduction of 50% already at a dose of 20 µg/ml [27]. Reduction of the absorption of plasma proteins on the particles’ surface is reported to prolong the blood circulation time of such nanoparticles drastically [28,29]. It is described that the amount of proteins detected on poly(lactic acid) (PLA) NPs functionalized with PEG was reduced to 57% of the amount adsorbed on pure PLA particles [28]. A recent study investigating the biodistribution of magnetic nanoporous silica nanoparticles in mice in an implant model via PET/CT and gamma counting reported that PEGylation resulted in lower liver and spleen uptake. However, significant differences in nanoparticle accumulation at an implantation site could not be revealed [22]. Another study with repeated injections of PEG-conjugated substances showed a so-called “accelerated blood clearance (ABC)” phenomenon, which resulted in a rapid clearance of the second dose of the injected substance [30]. The description of this phenomenon raised concerns about the safety of PEGylated nanoformulations and other PEG-modified drugs and led to intense research to find alternative functionalization.

The modification with CD-47, an integrin-associated protein (IAP) which is a universally expressed member of the immunoglobulin superfamily and plays an important role in self-recognition [31], is a promising approach to overcome limitations related to the functionalization of NPs with PEG by utilizing a different mechanism of action. CD-47 inhibits phagocytic uptake by interacting with the macrophage receptor SIRPα [32,33]. Phosphorylation of SIRPα’s cytoplasmic immunoreceptor tyrosine-based inhibitory motif (ITIM) and activation of phosphatases SHP-1 and SHP-2 are mechanisms involved in the inhibition of macrophage uptake [34,35]. Various literature already describes prolonged circulation times for NPs functionalized with CD-47 [36–38]. A study investigated the differences in blood circulation times for streptavidine-conjugated graphene nanosheets (SAGO) functionalized with either biotinylated PEG (BPEG) or biotinylated CD47-like SIRPα-binding peptide (BSP) after systemic administration in Balb/c mice. They found that the blood levels of BSP-SAGO after 8 hours were 2.5-fold higher than those for BPEG-SAGO nanosheets [36].

In the presented study magnetic nanoporous silica nanoparticles (MNPSNPs) were functionalized either with PEG or murine CD-47. To mimic an implant infection scenario, mice received previously infected magnetic and non-magnetic implants. The aim of this study was to compare organ as well as implant accumulation of the two differently modified nanoparticles *in vivo* in a mouse model of implant infection. It was hypothesized that an accumulation of CD-47 nanoparticles in the liver and spleen is lower compared to the PEGylated ones and with that, a higher accumulation on the implants and in the surrounding tissue is possible. Additionally, a higher accumulation of magnetic nanoparticles around the magnetic implant compared to the paramagnetic control was expected.

## Materials and methods

### Materials

For the syntheses, all chemicals were used without further purification. Iron(II) chloride tetrahydrate (≥99%), iron(III) chloride tetrahydrate (99%), oleic acid (90%), chloroform (≥99%), cetyltrimethylammonium bromide (CTAB, ≥ 98%), ammonium hydroxide solution (≥25% NH_3_ in water), tetraethyl orthosilicate (TEOS, ≥ 99%), ethyl acetate (99.8%), (3-aminopropyl)triethoxysilane (APTES, 99%), *N*-(3-dimethylaminopropyl)-N’-ethylcarbodiimid-hydrochlorid (EDC, ≥ 99%), *N*-hydroxysuccinimid (NHS, 98%), ethanol (EtOH, absolute, EMPLURA^®^), rhodamin B-isothiocyanat (RITC, mixed isomers) were purchased from Sigma-Aldrich Corporation (München, Germany), mouse CD-47 protein (CD47, < 90%, Sino Biological) was purchased from Biozol Diagnostica Vertrieb GmbH (Eching, Germany). 2-(*N*-morpholino)ethanesulfonic acid (MES, 99%) was purchased from fisher scientific GmbH (Schwerte, Germany).

### Synthesis of MNPSNP

The magnetic core-shell nanoparticles were prepared by the same method as previously reported [39–41]. Briefly, magnetite NPs were synthesized by a co-precipitation method. Afterwards a porous silica shell was condensed around the hydrophobic magnetite core using TEOS, ethyl acetate, and ammonium hydroxide after a phase transfer to the aqueous phase using CTAB was performed. After calcination, a porous core-shell particle was obtained.

#### PEG functionalization of MNPSNP.

**Fig 1:**
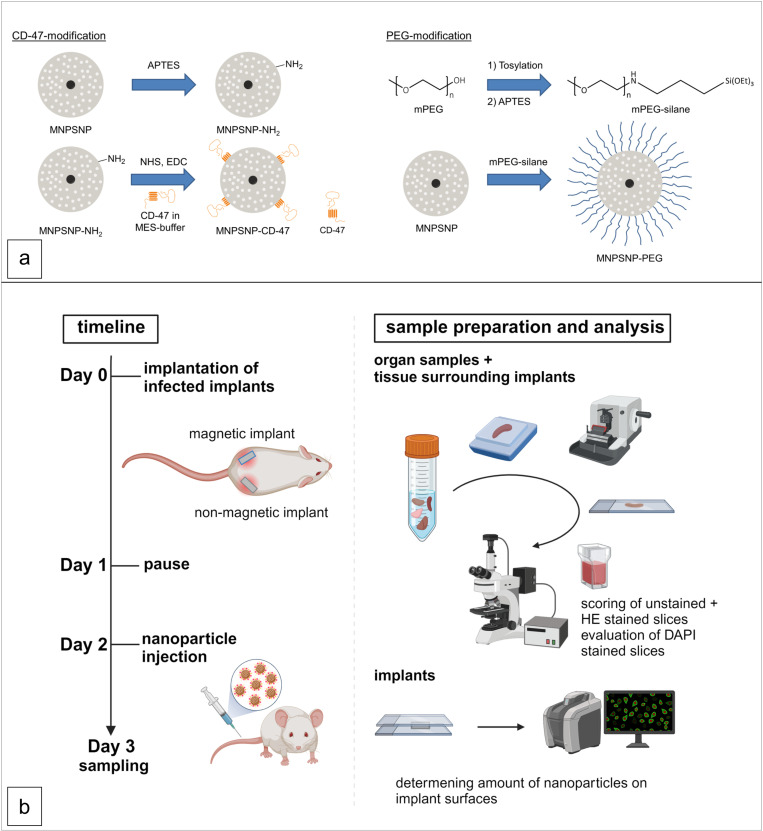
Schematic overview of nanoparticle synthesis (a) and timeline of animal study, sample preparation and analysis (b). Created with BioRender.com.

The synthesis of PEG-functionalized MNPSNP was carried out using a previously published method [41]. First, the mPEG-silane was synthesized starting from mPEG. After the tosylation of the alcohol and a subsequent reaction with APTES, the mPEG-silane was obtained. (Fig 1a)

This mPEG-silane was then attached to the surface of the MNPNP using a post-grafting method. Therefore, the MNPNSP were dispersed in ethanol and the mPEG-silane was added. To obtain fluorescent particles, also a previously synthesized RITC-silane was added to the synthesis. The synthesis was continued at 50°C for 24 h and after centrifugation and drying fluorescent PEG functionalized particles were obtained.

#### Amino functionalization of MNPSNP.

The amino functionalization of the synthesized MNPSNP, which is needed for protein immobilization was carried out by a similar method reported by Yuan et al [42]. In brief 150 mg of calcined MNPSNP were dispersed in 30 ml EtOH. Next, 1.5 ml APTES were added to the dispersion and the dispersion was stirred for 6 hours at 80 °C. After cooling, the MNPSNP were separated by centrifugation and washed three times with ethanol. The amino-functionalized MNPSNPs were dried under vacuum. (Fig 1a)

#### CD-47 immobilization.

CD-47 was dissolved in MES buffer (0.1 M, pH 5) in such way that a solution of 10 µg/ml was obtained. For the protein immobilization, 50 mg of amino functionalized particles, 28.5 mg NHS and 22 mg EDC were dissolved in 2.5 ml MES buffer (0.1 M) at pH 5. (Fig 1a) Next 2.5 ml of the CD-47 solution were added and the dispersion was shaken at 4 °C for 18 h. In the next step the particles were labelled using RITC. Therefore, the particles were centrifuged and after removing the supernatant 4 ml of a solution of 0.5 mg/ml RITC in water was added to the particles and the particles were further shaken at 4 °C for 3 h. Afterwards the particles were washed 4 times with 5 ml of water and dried under vacuum.

#### Characterization of the MNPSNP.

Transmission electron microscopy (TEM) was performed with a FEI Tencai G2 F20 TMP instrument (C_*S*_ = 2 mm, C_*C*_ = 2 mm) with a 200 kV field emission gun in bright-field mode at the Laboratory for Nano-and Quantum Engineering. For preparation, 400-mesh carbon-coated copper grids (Plano GmbH) were used. A small amount of the sample was taken out with the tip of a spatula dispersed in approx. 2 ml of ethanol via ultrasonication, dropped onto the grid and dried. If available, the size of 100 particles (at least 20 particles) was determined using NIH ImageJ and their size was averaged [43]. Zeta potential and dynamic light scattering (DLS) were measured with the Zetasizer Nano ZSP (Malvern Panalytical), wherefore a small amount of the sample was also taken out with the tip of a spatula. It was then dispersed in approx. 2 ml of water or FCS. From this dispersion 800 µl were transferred to a cuvette (DTS1070), measured three times and averaged. As a control, a sample of only FCS was measured. The pH of the used water was 6.9 and it did not change significantly after dispersing the nanoparticles. The hydrodynamic diameter and zeta potential were obtained by the associated software Zetasizer 7.11. Thermogravimetric analysis (TGA) was measured with a Netzsch STA 449 F5 Jupiter. The sample (10 mg) was heated to 1000 °C in an Al_2_O_3_ crucible with a rate of 5 °C min^-1^ in an atmosphere of N_2_ and O_2_ (80% N_2_, 20% O_2_). Nitrogen physisorption isotherms were measured on an Autosorb-3 System (Quantachrome/3P Instruments, Odelzhausen, Germany) at 77 K after outgassing 30 mg of the sample for 24 h at 110 °C under vacuum. Surface areas, pore sizes, and pore volumes were calculated using the associated software ASiQwin (Version 2.0) for the machine. Surface areas were calculated by applying the Brunauer-Emmett-Teller (BET) equation. The pore size distribution was determined by applying the non-linear density-functional theory (NLDFT) and fitting the Quantachrome Kernel “N2 at 77 K on silica for cylinder pores, NLDFT equilibrium model” to the experimental data. The values for total pore volumes were estimated by the single point method at p/p0 = 0.92 to exclude interparticular volume. All characterization methods were performed once on the respective sample, unless otherwise stated. However, the reproduction of all samples (each conducted at least three times) showed similar results. Additional characterization methods for the data displayed in the SI (FT-IR, VSM, and X-ray diffraction), are described there.

#### Detection of CD-47 and release experiment.

CD-47 was detected using the “Mouse CD47 ELISA Kit” from RayBiotech Inc. (Peachtree Corners, GA, United States). The ELISA was performed according to the instruction manual and the absorbance of the samples was measured using a Spark 10M from Tecan Trading AG (Mannedorf, Switzerland) at 450 nm.

For the release experiment, 2 mg of CD-47-modified particles without fluorescent dye were dispersed in 2 ml of phosphate-buffered saline at a pH of 7.4 and stored at 37 °C. At different time periods, the particles were centrifuged down, the supernatant was removed and fresh PBS was added. The particles were again dispersed and stored at 37 °C. The removed supernatant was stored at -20 °C until measuring its absorption at 450 nm three times. As a control the procedure was also carried out using amino functionalized particles.

### Animal model

*In vivo* experiments were authorized according to the German Animal Welfare Act and approved by the Lower Saxony State Office for Consumer Protection and Food Safety (registration number 33.19-42502-04-20/3394) and performed in 32 female BALB/CJRj mice with an average body weight (BW) of 22.56 g (± 1.76g). Mouse husbandry was organized in metal-free cages with a 14h/10h-day/night cycle and free access to food and tap water. Agarose gel was additionally provided when necessary.

All mice received a magnetic implant subcutaneously in the lateral femoral region on the left and a nonmagnetic implant on the right hind leg under general isoflurane anesthesia. (Fig 1b) The same implant material and surgical procedure as in Polyak et al. 2023 [22] was used. Implants had a size of 5 x 5 x 2 mm and were made either of grade 5 Ti-6Al-4V (non-magnetic implant) or neodymium (magnetic implant) with a thin titanium coating (Institute of Production Technology, Leibniz Universität Hannover). A grade 5 Ti-6Al-4V foil was purchased from Goodfellow GMBH, cut into the mentioned size by water jet cutting, and polished using a grinding machine with sandpaper. The implants were precultivated in a suspension containing S. aureus strain JSNZ wildtype (approximately 2.02 × 10^5^ CFU per ml TSB-Gluc) for 30 seconds prior to implantation as described in Reifenrath et al. 2020. The used S. aureus was provided and further described by Holtfreter et al [44]. After surgery mice received warmed isotone sodium chloride solution subcutaneously (5–10 ml/kg BW) and were placed under a heating lamp until recovery from anesthesia. During postoperative follow-up, mice were examined clinically every day. Pain relief was ensured by daily application of meloxicam (1 mg/kg BW s.c.). Nanoparticle application was performed on the second postoperative day. (Fig 1b) The mice were subdivided into two groups. One group received PEGylated and the other CD-47- functionalized nanoparticles. The person performing the applications was blinded to the nanoparticle type. Mice were anesthetized as described earlier and received 0.1 ml MNPSNP suspension intravenously into the tail vein (concentration of the nanoparticles in sodium chloride solution: 3.2 mg/ml). Mice again received warmed isotone sodium chloride solution subcutaneously and were placed under a heating lamp until recovery from anesthesia. 24 hours after nanoparticle application mice were sacrificed by cervical dislocation and intracardial blood sampling. Implants were explanted, put in 4% buffered formaldehyde solution for several minutes, and air-dried. A 3D-printed (Raise3D Pro2 Plus 3D-printer with dual extruder, 3Dmensionals/Pontialis GmbH&Co. KG, Germany) placeholder made of polylactic acide (RAISE3D Premium PLA Filament, 1.7 mm, 3Dmensionals/Pontialis GmbH&Co. KG, Germany) with dimensions like the implants was inserted to ensure later identification of the implantation site. Samples of the implantation region including the placeholder, lung, liver, spleen, kidneys, brain, and lymph nodes (Lnn. Iliaci) were taken and fixed in 4% buffered formaldehyde solution.

#### Detection of targeted enrichment of MNPSNPs on the implant surface.

Detection of the nanoparticles on the implant surfaces was performed according to Reifenrath et al. 2020 [21]. After air-drying magnetic and titanium implants were fixated between two microscope slides each and stored in darkness for later fluorescence microscopy. RITC-labeled nanoparticles were detected using a fluorescence microscope (Keyence BZ-X810, Keyence Deutschland GmbH, Neu-Isenburg, Germany) with a 100-fold magnification. Different red (BZ-X filter TRITC, model OP-87764, excitation 545/25 nm, emission 605/70 nm, Keyence Deutschland GmbH, Neu-Isenburg, Germany) and green fluorescent filters (BZ-X filter GFP, model OP-87763, excitation 470/40 nm, emission 525/50 nm, Keyence Deutschland GmbH, Neu-Isenburg, Germany) were used to detect corresponding areas. The pictures were matched afterwards to determine red fluorescent nanoparticles from attached autofluorescent cells and tissue. Overlay images were further analyzed with the freely available software ImageJ® [45]. The percentage of the red fluorescent area of the total implant surface was measured.

#### Histological evaluation of tissue slices.

The fixed samples of the inner organs as well as the tissue surrounding the implant were paraffin-embedded and cut in 5µm thin slices using a rotary microtome (Leica RM 2155, Leica Biosystems, Germany). The unstained tissue slices were analyzed using a fluorescence microscope (Axioskop 40, Carl Zeiss AG, Oberkochen, Germany). For nanoparticle detection a red filter (filter set 20, Excitatation BP 546/12, Beam Splitter FT 560, Emission BP 575–640, Carl Zeiss AG, Oberkochen, Germany) and for the control of autofluorescence a green filter (filter set 44, Excitation BP 475/40, Beam Splitter FT 500, Emission BP 530/50, Carl Zeiss AG, Oberkochen, Germany) was used. To evaluate the nanoparticle accumulation the same scoring system as described in Reifenrath et al. 2020 [21] was used. In brief, tissue slices were evaluated regarding the occurrence of nanoparticles and a score between zero (no nanoparticles detectable) and five (≥ 100 nanoparticle cluster detectable) was assigned. Hematoxylin and eosin (HE) staining of the tissue slices of the former implantation site was performed and slices were scored according to another scoring system also used in Reifenrath et al. 2020 [21] regarding inflammation, necrosis and fibrosis. In brief, the three parameters 1) presence and distribution of inflammatory reaction 2) presence and severity of fibrosis and 3) presence and distribution of necrotic debris were evaluated. Each parameter was scored according to score presented in S1 Table.

Corresponding 4′,6-diamidino-2-phenylindole (DAPI) stained slices of the tissue surrounding the former implantation site were investigated as well to evaluate whether more nanoparticle accumulation can be detected in areas with more severe signs of inflammation and cellular necrosis.

#### Statistics.

Statistical analysis was performed using GraphPad Prism 9 and SPSS 28 (IBM, USA). Tests were performed for the percentage of red fluorescent area of the total implant surface and the score values obtained for the unstained slices of the inner organs and tissue surrounding the implants. Wilcoxon test was chosen for the analysis of paired samples (left vs. right) and Kruskal-Wallis test was used to determine differences between groups (CD-47 vs. PEG). Spearmann´s correlation coefficient was calculated for analysis of the correlation between nanoparticle accumulation and severity of inflammation. Results were considered statistically significant for p ≤ 0.05.

### Results

#### Synthesis of MNPSNP.

**Fig 2:**
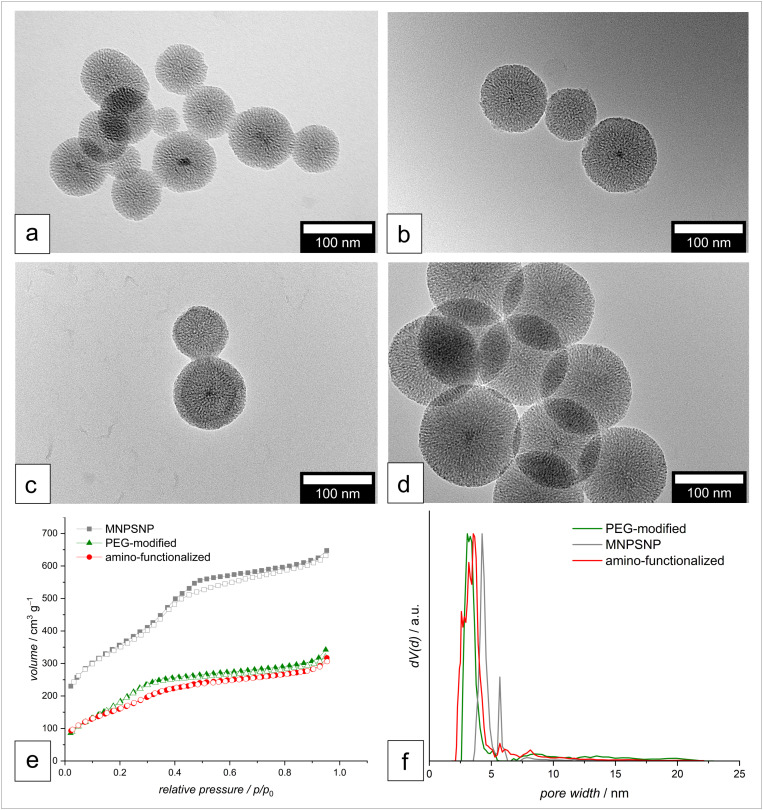
TEM-images, Nitrogen physisorption isotherms and pore size distribution of MNPSNP. TEM-images of MNPSNP before modification (a) and after amino functionalization (b) after PEG-modification (c) and CD-47-modification (d). Nitrogen physisorption isotherms (e) and pore size distribution (f) of MNPSNP, amino-functionalized MNPSNP, and mPEG-modified MNPSNP scale bar = 100 nm.

In the main manuscript, the results concerning the size, porosity, and surface charge of the modified MNPSNP are discussed. Further insights into the physical-chemical properties of the MNPSNP are shown in the SI (S1 Text: general information on additional data; S1 Fig., S2 Text: X-ray diffraction patterns; S2 Fig.: non-normalized pore size distribution; S3 Fig., S3 Text: FT-IR and S4 Fig.: TGA and VSM). Fig 2 presents the TEM images of the MNPSNP before and after PEG- or CD-47-modification, as well as the intermediate amino-functionalized MNPSNP. It is evident that the modification of the particles did not affect the shape and size of the particles. The MNPSNP before modification demonstrate a spherical shape with a diameter of 103 nm ± 14 nm, which remains similar after amino-functionalization (**⌀ =** 127 nm ± 12 nm) and after PEG modification (**⌀ =** 102 nm ± 12 nm) or CD-47 modification (**⌀** = 97 nm ± 11 nm). Thus, after both modifications the particles preserve their morphology (CD-47-modified particles were measured in limited quantities in these images). However, it is important to note, that fewer pores are accessible, as shown by the reduced values for inner surface area and pore volume determined by nitrogen physisorption measurements (Fig 2e, & Table 1). The calculated values for BET surface area, pore size, and pore volume are displayed in Table 1. The core-shell MNPSNP reveal with 1200 m^2^ g^-1^ a typical high surface area for nanoporous materials. After amino-functionalization, this value is decreased to 639 m^2^ g^-1^ presumably by the pore-blocking effect of grafted amino groups close to the pore entrances. Making the pore volume no longer accessible. The same effect is evoked by the attached PEG chains leading to a surface area of 725 m^2^ g^-1^. In Fig 2 f the pore size distributions of the corresponding particles are shown. The non-normalized distribution curves can be found in S2 Fig. in the SI. (Measurements on CD-47-functionalized MNPSNPs could not be performed due to low sample availability.)

**Table 1:** Values for the specific surface area (*S*_BET_) pore diameter (*D*_pore_) and pore volume (*V*_pore_) for pure, amino-functionalized, and mPEG-modified MNPSNPs, obtained from nitrogen physisorption isotherms. Values for the hydrodynamic diameter (D_*h*_), PDI and zeta-potential obtained from the dynamic light scattering experiments.

	*S*_BET_/ m^2^ g^-1^	*D*_pore_/ nm	V_pore_/ cm^3^ g^-1^	D_*h*_/ nm	PDI	Zeta-potential/ mV
MNPSNP	1200	4.0	0.9	103 ± 11	0.42 ± 0.11	-23
amino-functionalized	639	3.6	0.4	130 ± 22	0.65 ± 0.10	-14
mPEG-modified	725	3.4	0.4	141 ± 11	0.22 ± 0.01	-10
CD-47-modified	n/a	n/a	n/a	169 ± 49	0.76 ± 0.10	-12

**Fig 3:**
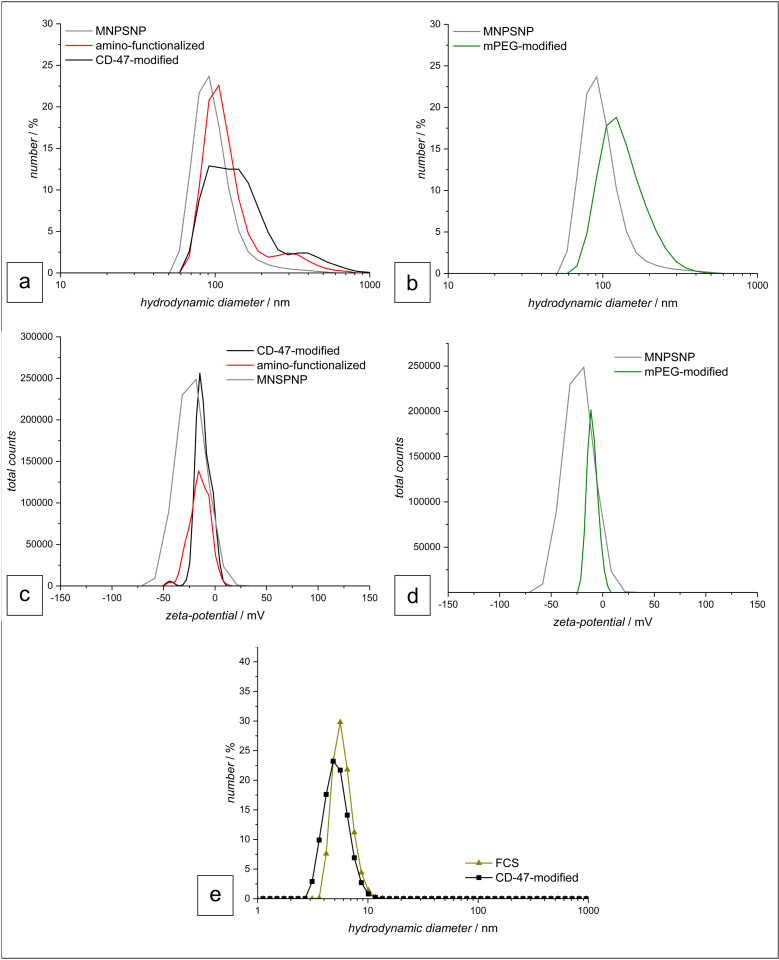
Size distribution obtained by dynamic light scattering. Particle size distribution of the MNPSNPs in the pathway of CD-47 modification **(a)**. Particle size distribution of the MNPSNPs in the pathway of mPEG modification **(b)**. Zeta potential distribution of mPEG-modified (c) and CD-47-modified MNPSNP with their respective pre-stages **(d)**. Size distribution of the hydrodynamic diameter of CD-47-modified MNPSNP in FCS with FCS for reference **(e)**.

The diameter of the particles was further investigated using dynamic light scattering (DLS) in water (Fig 3, Table 1). Here the hydrodynamic diameter is determined, which not only measures the particle size, but also takes the spatial arrangement of attached solvation molecules on the surface of the particles into account. Consequently, this typically results in a larger particle size compared to TEM measurements and reflects the diameter in an aqueous medium.

For both particles, mPEG and CD-47-modified, a size increase is observable compared to the unmodified MNPSNP. The latter exhibit a hydrodynamic diameter of 103 nm ± 11 nm, changing to a value of 130 nm ± 22 nm for the amino-functionalized particles in the progression of the particle synthesis. The CD-47-modified particles show a slightly higher diameter with 169 nm ± 49 nm, compared to the PEG-modified particles with 141 nm ± 11 nm. This larger diameter of the CD-47-modified particles is attributed to the higher spatial expansion of the protein and higher molecular mass (16.8 kDa) of the CD-47 relative to the PEG (2 kDa). In the DLS curves, a large size distribution can be found for both particle species, but it is more noticeable for the CD-47 particles. This indicates an agglomeration of the particles during the measurement, which is also visible in the large error value. This trend towards agglomeration also is confirmed in their respective zeta potential. Here, the different modified particles show a deviating zeta potential compared to the unmodified (-23 mV), which are significantly below the typical threshold of ± 30 mV, where a particle dispersion is considered stable. The CD-47-modified particles exhibit a zeta potential of -12 mV and the PEG of -10 mV. The distribution of the zeta potential can be found in Fig 3c and d. Here, the total counts of the sample across varying zeta potential values are shown. Since each count represents the population of particles exhibiting a specific zeta potential, this graph provides an overview of the zeta potential within the suspension. As for the experiments fetal calf serum (FCS) was used, the stability of CD-47-modified particles in this medium was investigated. Here, only similar values of the particle diameter to that of the pure FCS are obtained with single maxima at 5 nm, as shown in Fig 3e. The signal is mainly caused by the large amounts of proteins present in the FCS, which suppress the particle signal. In addition, the zeta potential is similar to the one of pure FCS with approx. -6 mV, precluding any definitive conclusions regarding particle stability in this medium. For this reason, no investigations with the PEG-modified particles in FCS were performed.

#### Determination of the immobilized amount of CD-47.

The amount of immobilized CD-47 on the MNPSNPs was indirectly determined by measuring the residual CD-47 in the supernatant after the attachment reaction, assuming the deficit represents the CD-47 bound to the particle surface. An additional immobilization experiment, scaled down by a factor of 2.5 from the method described in the experimental section and without fluorescent dye was carried out. Based on the data provided in S3 Tab. in the SI, an immobilization of 0.486 µg CD-47 per mg MNPSNP was revealed, which corresponds to an immobilization efficiency of 97%.

#### Stability test of attached CD-47 by release experiments.

**Fig 4:**
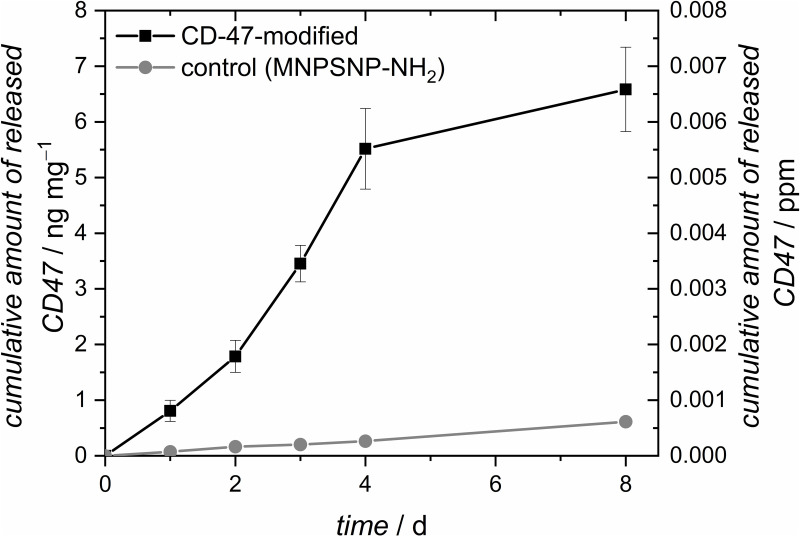
Release curve of CD-47-modified MNPSNPs (black) and amino-functionalized MNPSNP (grey) as control (MNPSNP-NH_2_).

To evaluate the stability of CD-47 immobilization, a release experiment was conducted with CD-47-modified particles in PBS over 8 days. The resulting release profile is illustrated in Fig 4 (black). It needs to be mentioned, that the individual released amounts are close to the detection limit of the used ELISA-kit (0.00045 ppm resp. 0.45 ppb). Throughout the experiment, only a minimal amount of the protein was released, totalling 5.2 ng mg^-1^ after 8 days. This release corresponds to less than 1% of the assumed total attached amount. Consequently, the attachment of CD-47 is deemed stable under the tested conditions. To check for any cross-reactivity of the ELISA with the nanoparticles, the release-experiment was also carried out using amino functionalized particles (MNPSNP-NH_2_). In this release curve (Fig 4, grey) no CD-47 could be detected, as all measurement points are below the detection limit of the used ELISA-kit (0.45 ng ml^-1^).

### Animal model

All animals showed a fast recovery from anaesthesia. Some animals had loosened stitches the afternoon after surgery, which were revised successfully. Mild swelling and reddening of the wounds were seen in the postoperative follow-up, with no other clinical changes observable. Hind limb mobility was not restricted by the implants. None of the mice lost more than five percent of their initial body weight in the days after surgery. The data of 27 animals was used for final evaluation and statistical analysis. Five animals, two from the CD-47 and three from the PEG group, dropped out due to no detectable nanoparticles in any tissue. A failed or uncertain nanoparticle injection was suspected. No association with the nanoparticle type could be seen.

#### Accumulation of MNPSNPs on implant surfaces.

**Fig 5:**
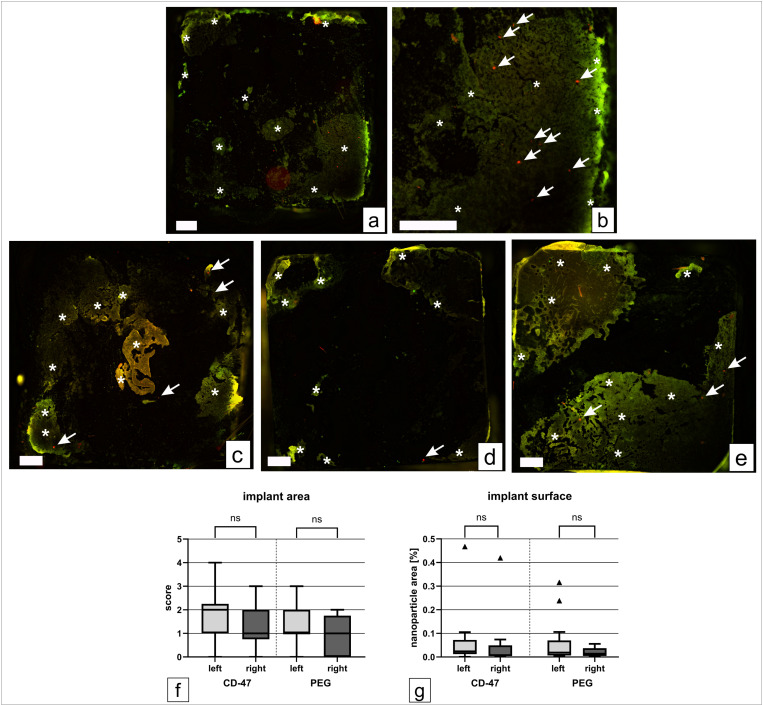
Exemplary fluorescent microscopy images of magnetic implants, fluorescence score values for the tissue surrounding the implants and percentage of red fluorescent area of the total implant surface. Implant of an animal from the CD-47 group (a), enlarged section of (a) with green autofluorescent material (b, asterisks) and red fluorescent nanoparticles (b, arrows); magnetic implant of an animal from the PEG-group (c); titanium implant of an animal from the CD-47 group (d); titanium implant of an animal from the PEG-group (e). Varying parts of the implants are covered with autofluorescent material (asterisks) which resulted in exclusion of some implant surfaces, the quantity of nanoparticles differs on the different implant materials and for the different nanoparticle types but without statistical significance, scale bar = 500 µm. Box and whisker plots of fluorescence score values for the tissue surrounding the implants (f) and the percentage of red fluorescent area of the total implant surface (g) for the two groups and the different implant materials (left = magnetic implant, right = non-magnetic implant). The boxes represent the 25^th^ to 75^th^ percentiles, the solid line indicates the median, triangles mark values outside the inner fences, CD-47 group n = 14 (both sides), PEG-group n = 13 (left) and n = 12 (right).

To determine the accumulation of nanoparticles on the implant surfaces an examination via fluorescence imaging was carried out (Fig 5). For the CD-47-group 14 implants and for the PEG-group 13 implants were included for statistical evaluation. Some implant surfaces had to be excluded due to a high amount of adhesive tissue which resulted in cellular autofluorescence. When both implant sides could be evaluated the mean was used for statistical analysis. Nanoparticles were detectable on both magnetic and non-magnetic implants. The nanoparticle amount which was calculated as the red fluorescent area of the implant was below 0.1% for most of the implants. (Fig 5g) We could not find any significant differences between the two nanoparticle types. There is a slight trend for a higher accumulation of nanoparticles for the magnetic implants, which were implanted on the left side. (Fig 5g)

#### Histological evaluation.

**Fig 6:**
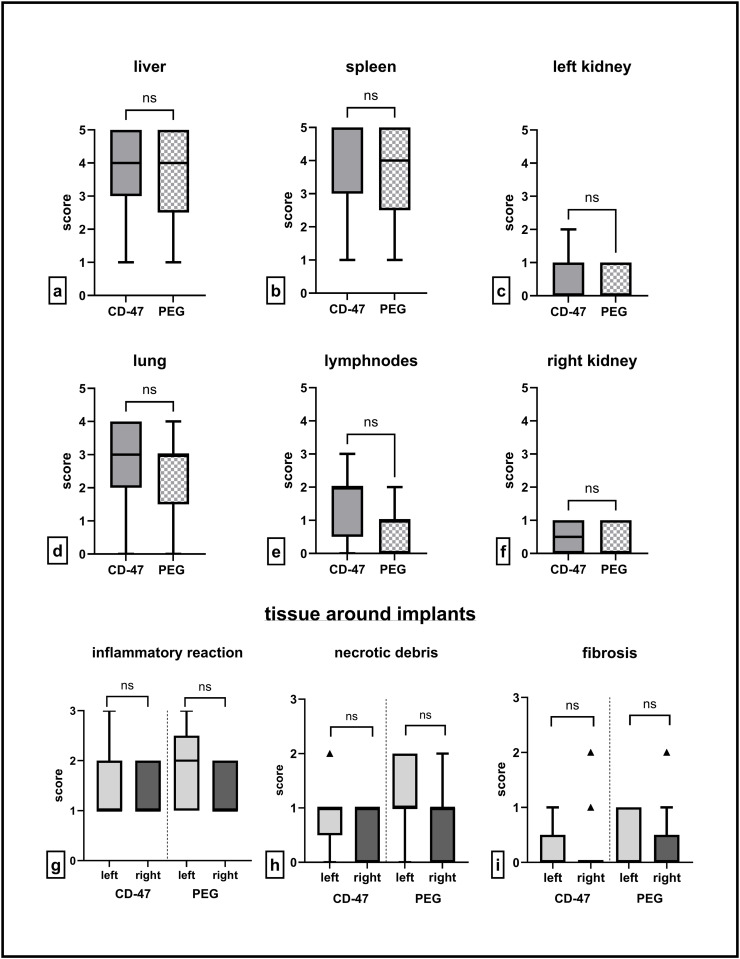
Box and whisker plots of the fluorescence score values of the inner organs and the tissue surrounding the implants. Score values of liver (a), spleen (b), left kidney (c), lung (d), lymphnodes (e) and right kidney (f) as well as score values for inflammatory reaction (g), necrotic debris (h) and fibrosis (i) of the tissue surrounding the implants for the two different groups. The boxes represent the 25^th^ to 75^th^ percentiles, the solid line indicates the median, triangles mark values outside the inner fences, CD-47 group n = 13 (left) and n = 11 (right), PEG-group n = 13 (both sides).

**Fig 7:**
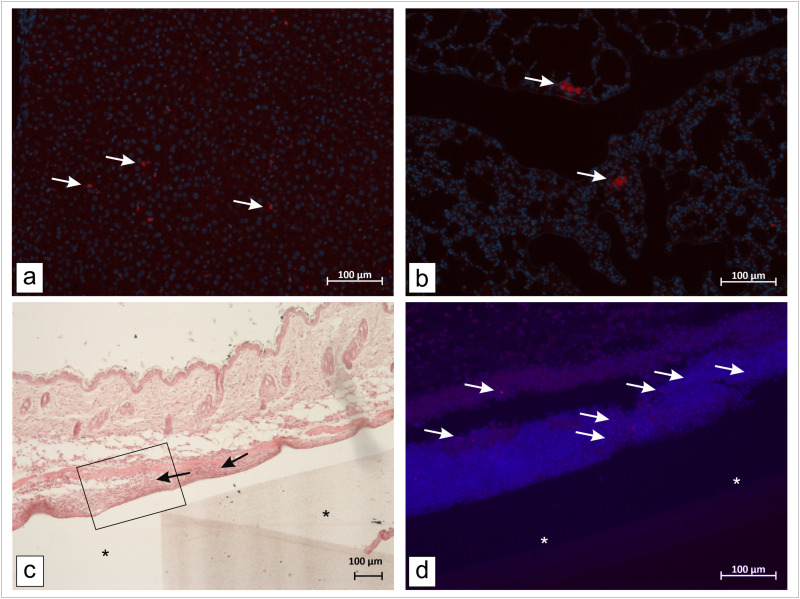
Exemplary light and fluorescence microscopic images of liver, lung and implantation site. Fluorescence microscopic images of smaller more solitary nanoparticles in the liver (a) in contrast to bigger clusters of nanoparticles in the lung tissue (b). For this animal (CD-47-group) a score of 4 (moderate, ≥ 100 cluster) for nanoparticles in the lung and a score of 5 (to a high degree, ≥ 100, confluence) for nanoparticles in the liver according to Reifenrath et al. [21] was assigned, arrows mark RITC-labeled nanoparticles; Light microscopic image of HE stained slice of the former implantation site with inserted placeholder (*) of an animal from the PEG-group showing infiltration of inflammatory cells (black arrows), for this slide a score of 2 for inflammatory reaction, 1 for necrotic debris and 1 for fibrosis was assigned (c) fluorescence microscopic image of DAPI stained slice of the same former implantation site like in c (black box in c **)**, RITC-labeled nanoparticles in areas with infiltration of inflammatory cells (white arrows) (d) ; blue: nuclear staining with DAPI, red: RITC labeled MNPSNP; scale bar = 100 µm.

The highest scores for nanoparticle accumulation were detected in liver (median of 4) and spleen (median of 5 for CD-47 and median of 4 for PEG) for both nanoparticle types. (Fig 6) No significant differences were found between the two nanoparticle types. With a median score of 3 the accumulation of NPs in the lung was also not negligible. In some animals, bigger clusters of nanoparticles were found in the lung tissue. (Fig 6 and Fig 7b) Regarding the implantation site a median score of 1 was assigned for both nanoparticle types around the non-magnetic implant. Around the magnetic implant a slightly higher median score of 2 was reached for the CD-47-modified nanoparticles compared to the median score of 1 for the PEGylated nanoparticles. (Fig 5f)

In almost all examined HE-stained slices the inserted placeholder allowed an easy identification of the former implantation site. (Fig 7c) Systematic scoring revealed varying degrees of inflammatory reaction with a median score of 1 for both implant types in the group which received CD-47-modified nanoparticles. In the PEG-group, a median score of 2 was assigned for the area around the magnetic implant, and a median score of 1 for the area around the non-magnetic implant. (Fig 6g) Regarding the presence and distribution of necrotic debris, a median score of 1 was given for both groups and implant materials. (Fig 6h) Only in some animals, signs of fibrosis were detectable resulting in a median score of 0 for both groups and implant materials. (Fig 6i) Significant differences in inflammatory reaction, presence and distribution of necrotic debris and severity of fibrosis were seen neither for the two different implant materials nor for the different nanoparticle variants. (Fig 6)

The DAPI-stained slices of the tissue surrounding the implants revealed that in most of the areas with stronger signs of inflammation, a higher number of nanoparticles was detectable. (Fig 7d) Nanoparticles mainly accumulated in areas with a higher degree of infiltration with inflammatory cells and in areas with cell death and necrosis. This was proved by statistical analysis of correlation between the fluorescence scores and the scores for inflammatory reaction, fibrosis, and presence of necrotic debris for the tissue surrounding the different implants. Positive correlations were only found for the left implant area, which was the side with the magnetic implant. For the CD-47-modified particles, a strong positive correlation was found for the number of nanoparticles around the former magnetic implant and the score for inflammatory reaction. (S2 Tab.) That was not the case for nanoparticles around the non-magnetic implant and inflammatory reaction. For the particles functionalized with PEG, no such correlation could be found. In contrast, in the PEG-group a strong positive correlation was found for the number of nanoparticles around the former magnetic implant and the score for necrotic debris. (S2 Tab.) When a sum score for all inflammation-associated scores is calculated a positive correlation can be seen between the sum score and the number of nanoparticles around the former magnetic implants for both nanoparticle types. (S2 Tab.)

### Discussion

Establishing new therapeutic options for implant-related infections is an extremely important topic. Carrying antibiotics directly to an infected implant using MNPSNPs might be a promising approach to decrease antibiotic concentrations needed and would allow the drug to develop its efficacy directly on site. Nevertheless, the interaction of the nanoparticles with the host´s immune system is an important problem and further improvements are needed. Within the study presented here, the functionalization of magnetic nanoporous silica nanoparticles with murine CD-47 is described and their organ- and implant-related accumulation compared to PEGylated nanoparticles *in vivo* in a mouse model of implant infection was investigated.

To overcome this major limitation a functionalization with CD-47 was chosen to improve nanoparticle accumulation on magnetic implants. Although it was possible to detect nanoparticles on the implants and in the tissue surrounding them the overall count was very low and no significant differences were found regarding the different nanoparticle types. These unexpected findings are in stark contrast to various reports in the literature.

CD-47 is a transmembrane protein which prevents cells from phagocytosis because it binds to signal regulatory protein-α (SIRPα), a protein expressed on macrophages, dendritic cells, granulocytes, monocytes and neurons [46], initiating a signaling cascade which inhibits phagocytosis [47–49]. It has been already shown that using the CD-47 SIRPα axis to prolong the circulation time of nanocarriers *in vivo* is possible. A study compared the blood circulation time of graphene oxide (GO) nanosheets functionalized with PEG or CD-47 like SIRPα binding peptide (SP) and showed that eight hours after the first injection blood levels of the SP functionalized nanosheets were 2.5-fold higher than those of the ones functionalized with PEG [36]. A more recent study evaluated the ability of CD-47 mimicry peptide coated liposomes to reduce macrophage-mediated clearance compared with PEGylated counterparts. The results revealed, amongst others, that the circulation time of the CD-47 mimicry peptide nanoliposomes was 1.5 times higher than that of the PEGylated formulation as well as reduced accumulation in the spleen, liver, heart and kidney tissue [50]. In the study presented here, nanoparticles accumulated mainly in the liver and spleen no matter which functionalization was applied, which was rather unexpected and is in stark contrast to the previously mentioned studies. When administered intravenously nanoparticles rapidly interact with diverse blood components for example immunoglobulins, blood-clotting factors, and complement proteins [51,52]. These interactions enable the formation of a protein corona, which is a prerequisite for the recognition by phagocytic cells of the mononuclear phagocytic system (MPS) like macrophages [47,53]. PEGylation and CD-47 functionalization provide different approaches to hinder recognition by the host’s immune system. The chains of polyethylene glycol attached to the nanoparticles` surface should hinder molecules from binding to the particles` surface. In contrast to that, CD-47 cannot hinder the interaction with blood components but when getting in contact with macrophages binding to SIRPα should inhibit phagocytosis. Due to that fact, a lower accumulation of CD-47-functionalized nanoparticles in liver and spleen and a higher accumulation at the implantation site of the magnetic implant was suspected but could not be proved. A possible explanation is an instability of the binding of the CD-47 to the nanoparticles. To test the stability of the nanoparticles *in vitro* a release experiment was conducted to evaluate the stability of the CD-47-modified particles in PBS over eight days, which revealed only minimal protein release indicating a stable attachment of the CD-47 under these conditions. Due to the disappointing *in vivo* results an additional investigation of DLS and zeta-potential of particles in FCS was carried out. Unfortunately, DLS- and zeta potential measurements were not evaluable since the signal of the proteins, present in large quantities in the serum, interferes with the signal of the nanoparticles. (Fig 3e)

CD-47 itself is a relatively large molecule, appearing at a 70.000 Da position upon SDS-PAGE due to its hyperglycosylated structure [49] . According to that, it can be difficult to use the protein on the surface of nano-sized formulations. An insufficient number of bound CD-47 could be another explanation for the unexpectedly poor performance of the nanoparticles investigated here. Although CD-47 is now frequently used in nanoparticle research, studies don’t report on how much CD-47 molecules are needed to achieve satisfying reduction of particle accumulation in cells of the MPS. To indirectly evaluate the amount of CD-47 immobilized on the MNPSNPs residual CD-47 in the supernatant after the attachment reaction was measured and an immobilization efficiency of 97% was revealed. (S3 Tab.) According to that, a high amount of CD-47 was bound to the particles. However, in the light of the poor performance of the particles, this result has to be interpreted carefully. No assumption can be made whether all of the protein was actually bound to the particles or if also some of the protein was destroyed during the attachment process. Furthermore, this method cannot be used to determine how the CD-47 molecules are distributed among the individual particles. Further examinations have to be performed to clarify this issue. According to the fact that no information could be found about the optimal number of CD-47 molecules bound to nanoparticles to prevent phagocytosis systematic experiments need to be conducted with varying numbers of molecules of CD-47 bound to the nanoparticles.

Both nanoparticle types were detectable directly on magnetic and non-magnetic implants as well as in the tissue surrounding them. A very interesting finding of the study presented here is, that in tissue surrounding implants with stronger signs of inflammation also a higher accumulation of nanoparticles could be seen, especially for the magnetic implants. A previous study using the same experimental setup already investigated the influence of macrophage depletion and infection on nanoparticle distribution in the body as well as the accumulation at an implant surface [21]. As expected they found significantly lower nanoparticle accumulation in the liver and spleen for the groups treated with clodronate liposomes because the injection of clodronate liposomes results in apoptotic cell death of macrophages mainly in the liver and to a lower degree also in the spleen [54,55]. Subsequently also a higher accumulation of nanoparticles around the implants was expected. In contrast to that, they found that the highest number of nanoparticles on implants was measured in the uninfected group without clodronate pretreatment followed by the infected group without clodronate treatment. They supposed that macrophages might play a crucial role in nanoparticle extravasation in the target area [21]. A recent study also highlighted the role of macrophages actively transporting nanoparticles in tumors after extravasation. They found that tumor-associated macrophages could carry nanoparticles 2–5 times deeper in the tumor than passive diffusion [56]. Whilst taking the higher number of nanoparticles in areas with more severe signs of inflammation into account, it might be possible that inflammation and involved immunocompetent cells like macrophages might play a crucial role in nanoparticle transport and their distribution in the tissue surrounding implants.

It is described in the literature that the microvascular structure and function is changed during the onset of inflammation when endothelial cells are activated by pro-inflammatory mediators, leading to vascular permeability [57,58] . Endothelial leakiness is described as one of the critical steps in the inflammation process. Arteriole dilation, an increase in venule permeability and release of chemical signals are part of the immediate non-targeted response towards foreign bodies like bacteria or viruses [58]. Macrophages and other white blood cells exploit the vessel leakiness and leave the vessels through these gaps [59]. This might be a possible explanation for the higher accumulation of nanoparticles in the tissue with stronger signs of inflammation. Additionally, nanoparticles already engulfed in macrophages or granulocytes might be brought to the infection site at the implant. Next to nanoparticle uptake by macrophages, uptake by granulocytes was also described as one of the main reasons for fast nanoparticle clearance from the circulation in Balb/c mice [60].

Generally, the two main routes of nanoparticle transport across the endothelium are the transcellular and the paracellular route [61]. NPs enter endothelial cells via endocytosis and are transported intracellularly via the transcellular route, whereas in the paracellular route intercellular gaps are needed for the particles to penetrate through [62–64]. Under normal conditions the width of gaps between endothelial cells is just two to six nanometers. It was observed that the interaction of NPs with the endothelium widens existing gaps or induces new ones in the monolayer of endothelial cells [64,65].

Regarding the results of the study presented here, it seems that the used magnetic nanoporous silica nanoparticles with a size above 100 nm might not have been able to induce an endothelial widening. Further investigations are needed to better understand the exact mechanisms involved in extravasation of this kind of nanoparticles in an inflammatory environment.

Another rather unexpected finding of the current study was the accumulation of particle clusters in the lung especially for the CD-47 nanoparticles. (Fig 7) Another possible explanation for this and also the high accumulation in the liver and spleen might be an agglomeration of the particles. It is well known that physiochemical properties like size, charge and surface chemistry affect the biodistribution and clearance of nanoparticles [66,67]. A recent review by Kumar et al. investigating the biodistribution of a variety of nanoparticles by reviewing a total of 2018 datasets from various studies, reports that larger silica nanoparticles are majorly distributed to lung (12.1%ID/g), spleen (2.1%ID/g) and liver (19%ID/g) [68]. Regarding size, the upper critical limit is reported as a diameter of 200 nm [69,70] or 150 nm for spherical nanoparticles [24]. It was investigated that particles with a larger diameter activate the complement system resulting in a quick accumulation in the liver and spleen. The size of the nanoparticles used here, determined by DLS, was 141 nm ± 11 nm (PEG) and 169 nm ± 49 nm (CD-47), respectively, which is close to or above the reported optimal size range for spherical particles. This could be a possible explanation for the high accumulation rates in liver and spleen. As previously described, nanoparticles in agglomeration state show a higher cell uptake *in vitro* compared to non-agglomerated ones [71]. To enable the detection of the nanoparticles a functionalization with RITC was necessary, which might have had an impact on the formation of agglomerates. That’s why an additional experiment investigating the agglomeration of bare CD-47 modified MNPSNPS compared to RITC functionalized ones was performed. (S5 Fig.) This suggests that functionalization with fluorophores required only for imaging could negatively influence the physicochemical and biological properties of the NPs and that accumulation in the target tissue could be higher without them. Nevertheless, further studies are also required in this area.

CD-47 functionalization of nanoparticles is generally a promising approach to create so-called stealth nanoparticles. Nevertheless, within this study inflammatory cells and vessel leakiness seemed to be crucial for nanoparticle accumulation around infected implants independent of the applied functionalization.

### Conclusions

Although various literature describes higher accumulation rates for nanoparticles functionalized with CD-47 in target areas and a reduced accumulation in liver and spleen, this could not be shown within this study. With median score values of 4 (PEG & CD-47) for liver and 4 (PEG) and 5 (CD-47) for spleen, highest nanoparticle accumulation was detected in these organs. No significant differences were found regarding implant accumulation of PEG or CD-47-modified particles. A positive correlation between a sum score for inflammation-associated changes to the number of nanoparticles at the direct magnetic implant interface for both nanoparticle types indicates that inflammation-associated changes in tissue and vessels might be crucial for nanoparticle delivery to magnetic implants. Possible *in vivo* instability, an insufficient number of CD-47 molecules bound to the particles or rapid agglomeration of the particles might be plausible reasons for the unexpectedly poor performance of the CD-47 nanoparticles and the lack of significant differences compared to the PEG-functionalized NPs. As currently no information is available on the optimal number of CD-47 molecules bound to nanoparticles to prevent phagocytosis this should be investigated in future studies for example using macrophages *in vitro* to quantify the uptake of bare nanoparticles and nanoparticles functionalized with different numbers of CD-47 molecules. Additionally, it would be beneficial to get deeper insights into the interactions of CD-47-modified nanoparticles with proteins present in the blood. A combination of SDS-PAGE (sodium dodecyl sulfate polyacrylamide gel electrophoresis) with high resolution LC-MS (liquid chromatography-mass spectrometry) after incubation with FCS, as already described in the literature for PEG-functionalized nanoparticles [72], could be used to characterize the formed protein corona. Furthermore, investigations of the underlying mechanisms of the interaction of the used nanoparticles with endothelial cells should also be part of future studies to better understand their extravasation mechanism.

## Supporting information

S1 Text Information on the analytical data provided in the Supporting Information. (DOCX)

S1 Fig X-ray diffraction patterns of the (a) magnetite core material and (b) of the unmodified MNPSNP The grey shaded reflections in (a) belong to the used sample holder and can be neglected. In (a) the hkl-values for magnetite are assigned to the corresponding reflections.
(TIF)

S2 Text Information on the X-ray diffraction patterns in S1 Fig. (DOCX)

S2 Fig Non-normalized pore size distribution of (a) unmodified MNPSNP, (b) amino-functionalized and (c) mPEG-modified particles. Measurements on CD-47-functionalized MNPSNPs could not be performed due to low sample availability.
(TIF)

S3 Text Additional characterization of the organic content of the MNPSNPs by IR spectroscopy and thermogravimetric analysis. (DOCX)

S3 Fig IR-spectra of mPEG-modified (a) and CD-47-modified (b) MNPSNP and mass loss of mPEG-modified (c) and CD-47-modified (d) MNPSNP. All graphs contain the respective pre-stages of the modified particles.
(TIF)

S4 Fig Vibrating sample magnetometer (VSM) magnetization curve of MNPSNP and mPEG-modified MNPSNP. Both particles show a superparamagnetic behavior with no remanence. The saturation magnetization for the unmodified MNPSNP is 1 emu g^-1^ and 0.85 emu g^-1^ for the mPEG-modified MNPSNP.
(TIF)

S1 Table Scoring of inflammatory reaction, fibrosis and presence of necrotic debris around the former implantation site in paraffin embedded and HE-stained tissue slices. (DOCX)

S2 Table P- and Spearmanns´s ρ-values for the correlation between fluorescence score for the tissue surrounding the magnetic implant and score values for inflammatory reaction, necrotic debris, and a sum score of inflammatory reaction, necrotic debris, and fibrosis. (DOCX)

S3 Table Relative absorbance and calculated values of the supernatant and washing solution from the CD-47 immobilization. Using this data and the amounts used in the immobilization experiment (1 ml of a 10 µg ml^-1^ solution), the immobilized amount of CD-47 on 20 mg of particles was calculated to be 9.72 µg, which represents the 0.486 µg CD-47 per mg particle mentioned in the main text.
(DOCX)

S5 Fig Nanoparticles functionalized just with CD-47 (left flask), nanoparticles functionalized with CD-47 and RITC (middle flask) incubated in fetal calf serum (FCS, right flask). (a) undispersed (b) freshly dispersed (c) 2 min after dispersion (d) 6 min after dispersi**on,** nanoparticles with additional RITC functionalization seem to agglomerate faster in FCS than the particles without RITC.
(TIF)
