# Supplementary material for: Comparison of accumulation and distribution of PEGylated and CD-47-functionalized magnetic nanoporous silica nanoparticles in an in vivo mouse model of implant infection
Source: PLoS One. 2025 May 2;20(5):e0321888. doi: 10.1371/journal.pone.0321888 (PMC12047780; doi:10.1371/journal.pone.0321888)
Supplement: S1 Text — (DOCX) [file pone.0321888.s001.docx]

**S1 Text. Information on the analytical data provided in the Supporting Information**

Transmission electron microscopy, dynamic light scattering, zeta potential measurement, nitrogen physisorption and thermogravimetric analysis were performed as mentioned in the section “Characterization of the MNPSNP” in the main text. Fourier-transform infrared spectroscopy (FT-IR) was measured on a Spectrum Two FT-IR spectrometer from Perkin Elmer (Waltham, United States) using an ATR set-up, using a small amount of sample which was taken out with the tip of a spatula, in the area from 4000 cm^-1^ to 400 cm^‑1^. The magnetization curves of the samples were measured using the vibrating sample magnetometer Model 7407 (Lake shore Cryotronics Inc., Westerville, Ohio, USA). Approximately 10 mg of the sample was weighed out and its magnetization was measured in a field from -15 to 15 kOe. The X-ray diffraction pattern for the core material was recorded on a *θ/θ*-diffractometer (Stoe, Darmstadt, Germany) in reflection geometry using Cu*Kα* radiation (λ=1.540 Å) and a secondary beam monochromator (graphite). The sample was placed into the holder and the reflections were measured with a step size of 0.020 °*2θ* between 20 to 70 °*2θ* and a measurement time of 5 s per step. The pattern of the MNPSNP was recorded in transmission geometry using Cu*Kα* radiation (λ=1.540 Å) on a Stadi P powder diffractometer (Stoe & Cie GmbH, Darmstadt, Germany). The measurement was performed with a step size of 0.015 °*2θ* between 1 and 70 °*2θ* and a measurement time of 10 s per step.
All characterization methods were performed once on the respective sample, unless otherwise stated. However, the reproduction of all samples (each conducted at least three times) showed similar results.
