# Supplementary figures and images for: Comparison of accumulation and distribution of PEGylated and CD-47-functionalized magnetic nanoporous silica nanoparticles in an in vivo mouse model of implant infection
Source: PLoS One. 2025 May 2;20(5):e0321888. doi: 10.1371/journal.pone.0321888 (PMC12047780; doi:10.1371/journal.pone.0321888)

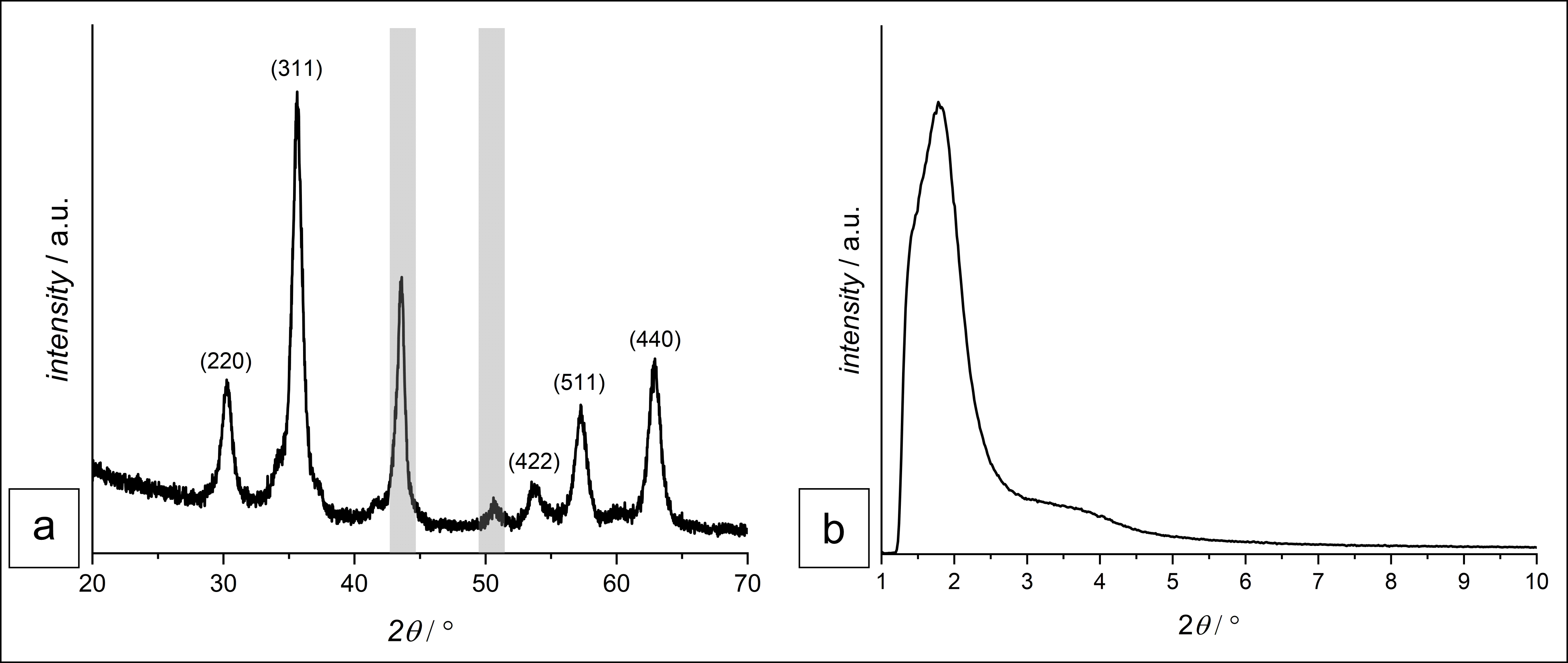

Supplement: S1 Fig — In (a) the hkl-values for magnetite are assigned to the corresponding reflections. (TIF) [file pone.0321888.s002.tif]

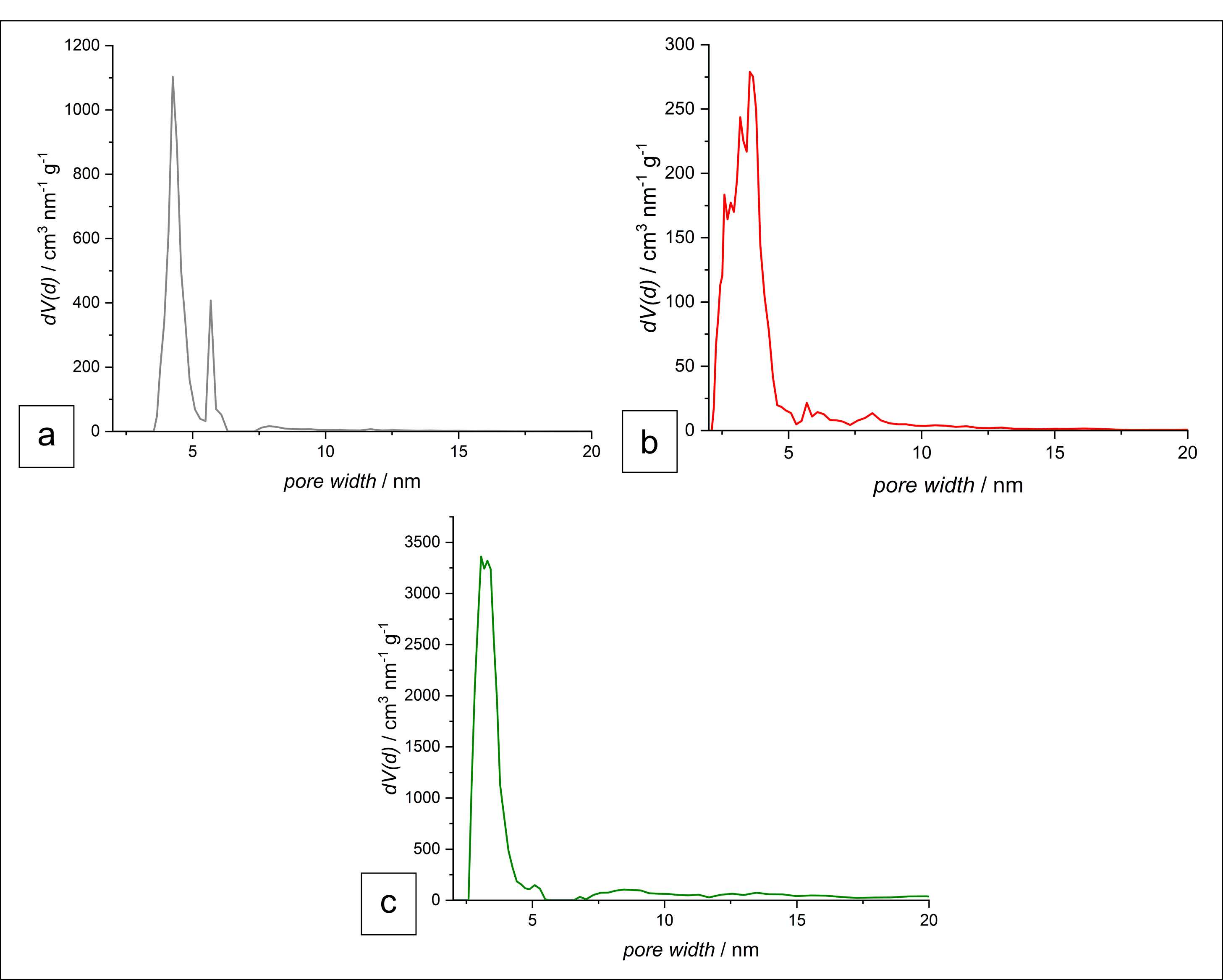

Supplement: S2 Fig — Measurements on CD-47-functionalized MNPSNPs could not be performed due to low sample availability. (TIF) [file pone.0321888.s004.tif]

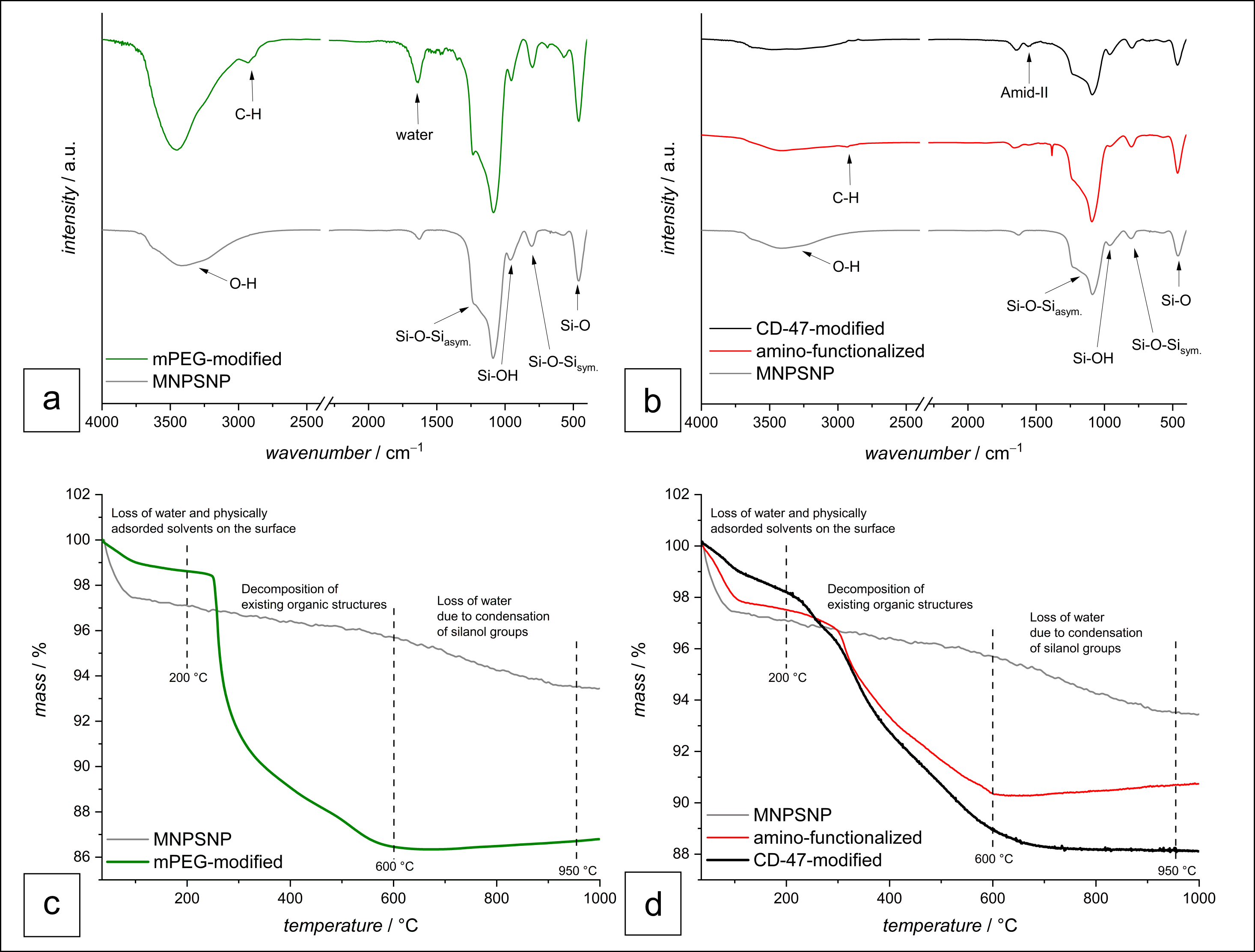

Supplement: S3 Fig — All graphs contain the respective pre-stages of the modified particles. (TIF) [file pone.0321888.s006.tif]

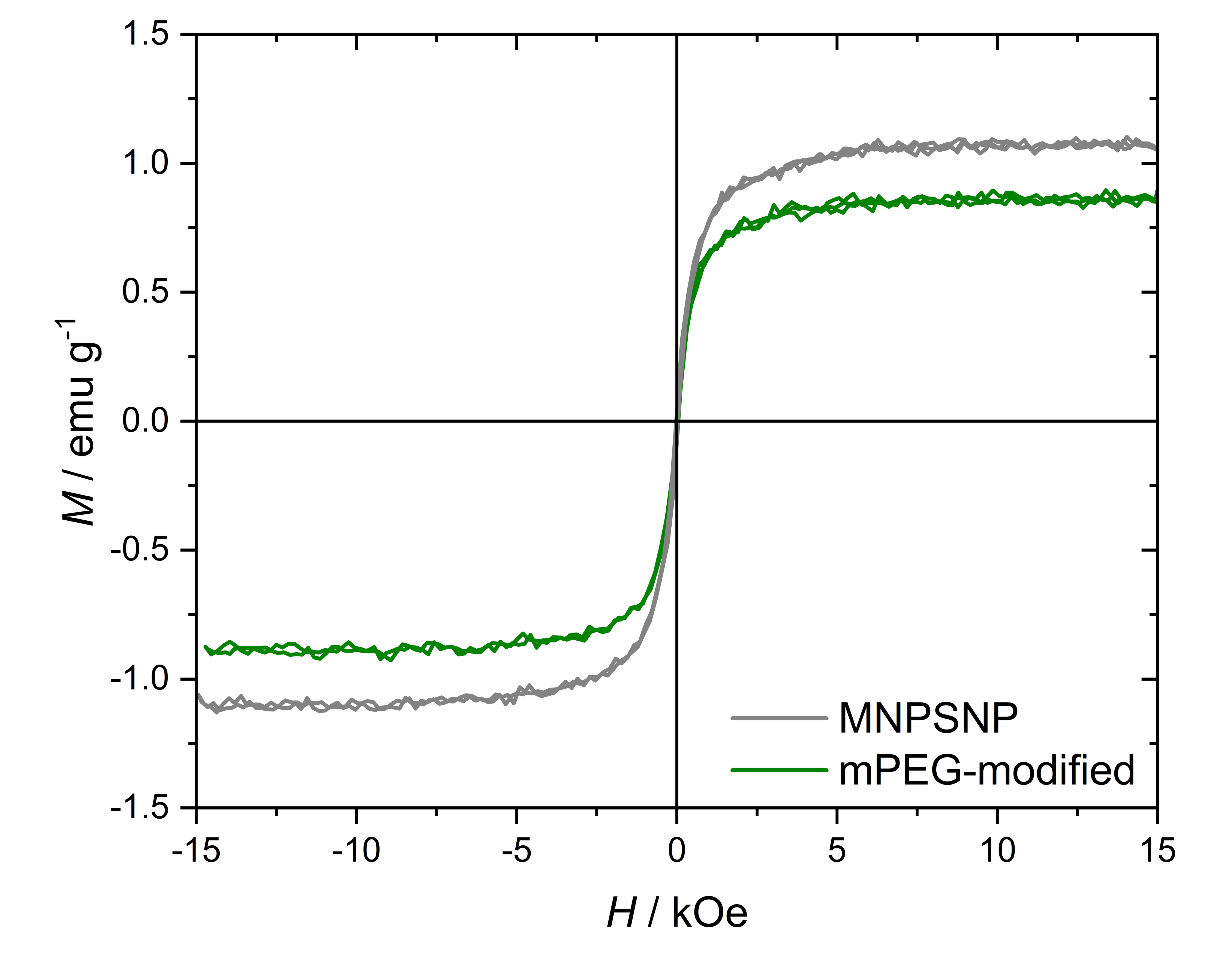

Supplement: S4 Fig — Both particles show a superparamagnetic behavior with no remanence. The saturation magnetization for the unmodified MNPSNP is 1 emu g-1 and 0.85 emu g-1 for the mPEG-modified MNPSNP. (TIF) [file pone.0321888.s007.tif]

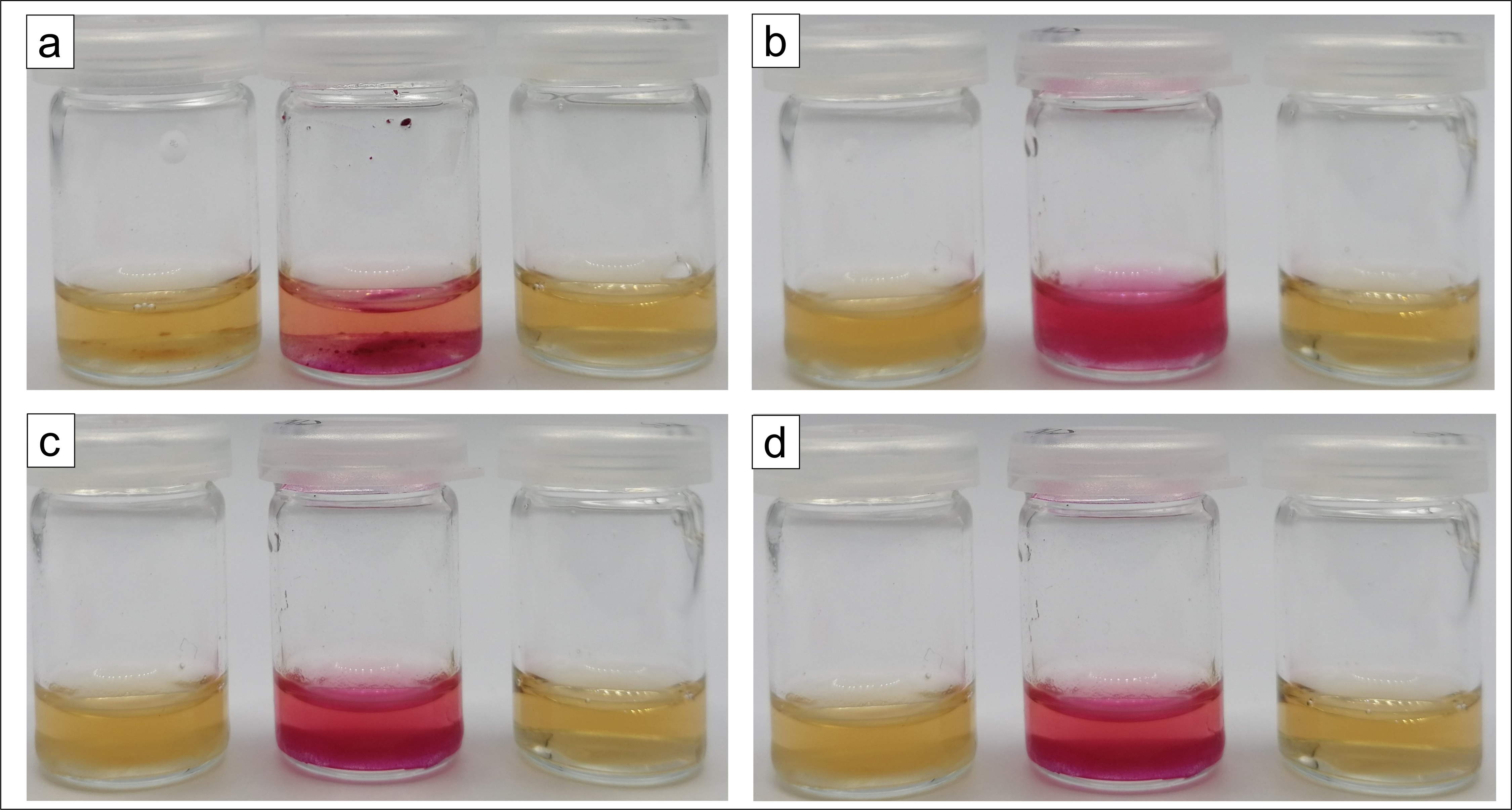

Supplement: S5 Fig — (a) undispersed (b) freshly dispersed (c) 2 min after dispersion (d) 6 min after dispersion, nanoparticles with additional RITC functionalization seem to agglomerate faster in FCS than the particles without RITC. (TIF) [file pone.0321888.s011.tif]
