# Supplementary material for: Comparison of accumulation and distribution of PEGylated and CD-47-functionalized magnetic nanoporous silica nanoparticles in an in vivo mouse model of implant infection
Source: PLoS One. 2025 May 2;20(5):e0321888. doi: 10.1371/journal.pone.0321888 (PMC12047780; doi:10.1371/journal.pone.0321888)
Supplement: S2 Text — (DOCX) [file pone.0321888.s003.docx]

**S2 Text. Information on the X-ray diffraction patterns in S1 Fig.**

In S1 Fig. the X-ray diffraction pattern of the core material and the unmodified MNPSNPs is shown. The pattern for the core material exhibits the typical reflections for a magnetite/maghemite material. The grey-shaded reflections belong to the used sample holder and can be neglected.
A single broad reflection at approx. 1.8 °*2θ* is observable for the MNPSNPs. This is caused by the electron density contrast between the silica wall and the empty pores. It confirms the disordered mesoporous structure of the MNPSNPs. Reflections in the X-ray diffraction pattern of the MNPSNPs, which could be assigned to the magnetite core, could not be observed. This is attributed to the small amount of core material present in the core-shell particle.
