# Supplementary material for: Comparison of accumulation and distribution of PEGylated and CD-47-functionalized magnetic nanoporous silica nanoparticles in an in vivo mouse model of implant infection
Source: PLoS One. 2025 May 2;20(5):e0321888. doi: 10.1371/journal.pone.0321888 (PMC12047780; doi:10.1371/journal.pone.0321888)
Supplement: S3 Text — (DOCX) [file pone.0321888.s005.docx]

**S3 Text. Additional characterization of the organic content of the MNPSNPs by IR spectroscopy and thermogravimetric analysis**

In S3 Fig. IR spectra and results of the thermogravimetric measurements of the modified MNPSNP with their respective pre-stages are shown. In the IR spectra, a clear change in present vibration bands is observable after each synthesis step. For the mPEG-modified MNPSNP, a clear C-H-vibration band is visible after the modification, which belongs to the attached PEG moiety (S3a Fig). The attached organic mass was further confirmed using thermogravimetric measurements, where a mass loss of 12 % was measured for the mPEG-modified particles (S3c Fig). Similar results are obtained for the CD-47-modified MNPSNP. Here, the amid-II vibration band is visible after the attachment of the protein (S3b Fig). This band is caused by the large amounts of amide bonds within the protein, which connects the amino acids in the primary structure. Here, also a higher mass loss compared to the unmodified MNPSNP with 10 % is observable, confirming the attachment of additional organic mass to the particle (S3d Fig.).
