# Supplementary material for: Comparison of accumulation and distribution of PEGylated and CD-47-functionalized magnetic nanoporous silica nanoparticles in an in vivo mouse model of implant infection
Source: PLoS One. 2025 May 2;20(5):e0321888. doi: 10.1371/journal.pone.0321888 (PMC12047780; doi:10.1371/journal.pone.0321888)
Supplement: S1 Table — (DOCX) [file pone.0321888.s008.docx]

**S1 Table**. **Scoring of inflammatory reaction, fibrosis and presence of necrotic debris around the former implantation site in paraffin embedded and HE-stained tissue slices.**

| **Score value** | **Occurrence of parameters** | **% of affected peri-implant interface** |
| --- | --- | --- |
| 0 | absent | no presence of any of the considered parameter |
| 1 | scarcely present | parameter detected on < 33% of the peri-implant interface |
| 2 | present | parameter found in 33 – 66 % of the peri-implant interface |
| 3 | Intensively present | parameter found in > 66% of the peri-implant area |
