# Supplementary material for: Comparison of accumulation and distribution of PEGylated and CD-47-functionalized magnetic nanoporous silica nanoparticles in an in vivo mouse model of implant infection
Source: PLoS One. 2025 May 2;20(5):e0321888. doi: 10.1371/journal.pone.0321888 (PMC12047780; doi:10.1371/journal.pone.0321888)
Supplement: S2 Table — (DOCX) [file pone.0321888.s009.docx]

**S2 Table. P- and Spearmanns´s ρ-values for the correlation between fluorescence score for the tissue surrounding the magnetic implant and score values for inflammatory reaction, necrotic debris, and a sum score of inflammatory reaction, necrotic debris, and fibrosis.**

|  | CD-47 group | | PEG group | |
| --- | --- | --- | --- | --- |
|  | **fluorescence score left implant area** | | **fluorescence score left implant area** | |
|  | Spearmanns´s ρ | p-value | Spearmanns´s ρ | p-value |
| inflammatory reaction left | 0.594 | 0.032 | - | - |
| necrotic debris left | - | - | 0.686 | 0.010 |
| sum change left | 0.683 | 0.007 | 0.552 | 0.050 |
