# Supplementary material for: Comparison of accumulation and distribution of PEGylated and CD-47-functionalized magnetic nanoporous silica nanoparticles in an in vivo mouse model of implant infection
Source: PLoS One. 2025 May 2;20(5):e0321888. doi: 10.1371/journal.pone.0321888 (PMC12047780; doi:10.1371/journal.pone.0321888)
Supplement: S3 Table — Using this data and the amounts used in the immobilization experiment (1 ml of a 10 µg ml-1 solution), the immobilized amount of CD-47 on 20 mg of particles was calculated to be 9.72 µg, which represents the 0.486 µg CD-47 per mg particle mentioned in the main text. (DOCX) [file pone.0321888.s010.docx]

**S3 Table.** **Relative absorbance and calculated values of the supernatant and washing solution form the CD47 immobilization.**

| relative absorbance | blank value | concentration of CD-47/ ng ml^-1^ | mass of CD-47  / ng |
| --- | --- | --- | --- |
| 1.88  (supernatant, 1:2 dilution) | 0.06 | 114.13  (w/o dilution) | 228.26  (w/o dilution) |
| 0.54  (washing solution) | 0.06 | 14.34 | 30.00 |
| 0.41  (washing solutions) | 0.06 | 10.86 | 21.71 |
